# Dataset of alkaline ethylene glycol pretreatment and two-staged acid hydrolysis using oil palm empty fruit bunch

**DOI:** 10.1016/j.dib.2020.105431

**Published:** 2020-03-17

**Authors:** Danny Wei Kit Chin, Steven Lim, Yean Ling Pang, Chun Hsion Lim, Kiat Moon Lee

**Affiliations:** a Department of Chemical Engineering, Lee Kong Chian Faculty of Engineering and Science, Universiti Tunku Abdul Rahman, 43000 Kajang, Selangor, Malaysia; b Centre of Photonics and Advanced Materials Research, Universiti Tunku Abdul Rahman, 43000 Kajang, Selangor, Malaysia; c Department of Chemical & Petroleum Engineering, Faculty of Engineering, Technology and Built Environment, UCSI University, 56000 Kuala Lumpur, Malaysia

**Keywords:** Lignocellulosic biomass, Alkaline organosolv pretreatment, Acid hydrolysis, Oil palm empty fruit bunch, Biofuel, Biochemical

## Abstract

Lignocellulosic biomass can provide a consistent and sustainable source of bioenergy. Degraded empty fruit bunch (DEFB) from oil palm is a suitable candidate for biofuels and biochemicals production by using alkaline ethylene glycol pretreatment and two-staged acid hydrolysis. This paper provided several important physical and chemical properties of treated DEFB after alkaline ethylene glycol pretreatment. The dataset for analysis methods which were used in two-staged acid hydrolysis for the determination of reducing sugars, phenolic compounds, furfural and hydroxymethyfurfural (HMF) in the acid hydrolysate was also provided. The key information and dataset provided in this article can be useful for researchers to determine the product compositions from lignocellulosic biomass after the pretreatment and acid hydrolysis processes in the future.

Specifications table

**Table:** 

Subject	Renewable Energy, Sustainability and Environment
Specific subject area	Lignocellulosic Biomass, Biofuels, Bioproducts
Type of data	Table, Image, Graph
How data were acquired	The data were acquired based on the analytical instrument which included scanning electron microscope (SEM), Fourier Transform Infrared Spectroscopy (FTIR), Electron Dispersive X-ray (EDX), X-ray Diffraction (XRD), High Performance Liquid Chromatography (HPLC) and UV–vis Spectrophotometry.
Data format	Raw and analysed.
Parameters for data collection	Characterisations of fresh empty fruit bunch (EFB), degraded EFB (DEFB) and treated DEFB after alkaline ethylene glycol pretreatment and dataset for analysis methods used for the acid hydrolysate obtained from the two-staged acid hydrolysis.
Description of data collection	The characterisations of fresh EFB, DEFB and treated DEFB were conducted by using SEM, EDX, FTIR and XRD. 3,5-Dinitrosaclicylic acid (DNS) and Folin Ciocalteus reagent tests were measured by UV–Vis spectrophotometry. Concentrations of furfural and hydroxymethylfurfural (HMF) were quantified by HPLC.
Data source location	Institution: Lee Kong Chian Faculty of Engineering and Science, Universiti Tunku Abdul Rahman (UTAR) City/Town/Region: 43,000 Kajang, Selangor Country: Malaysia
Data accessibility	The data were available in this article.
Related research article	Chin, D.W.K., Lim, S., Pang, Y.L., Lim, C.H., Lee, K.M., 2019. Two-staged acid hydrolysis on ethylene glycol pretreated degraded oil palm empty fruit bunch for sugar based substrate recovery. Bioresour. Technol. 292, 121,967. https://doi.org/10.1016/j.biortech.2019.121967. [1]

## Value of the Data

• The presented dataset are useful for researchers to study on different pretreatment methods for biofuel and biochemical production since they are still very rarely accessible in the literature.
• The various characterisations tests carried out in this study can serve as an important reference for other researchers intending to perform similar analysis.
• The characterisation results after alkaline ethylene glycol pretreatment can be used as a comparison with other pretreatment methods to develop an effective pretreatment for other types of lignocellulosic biomass.
• The dataset of the analysis methods for the acid hydrolysate can be used as reference for developments of advanced analysis methods to further improve their accuracy and consistency.
• The data in this article provide the transformation of DEFB after subjected to the alkaline ethylene glycol pretreatment and two-staged acid hydrolysis which are essential for researchers and scientific community in this field.

## 1 Data description

**Fig. 1:**
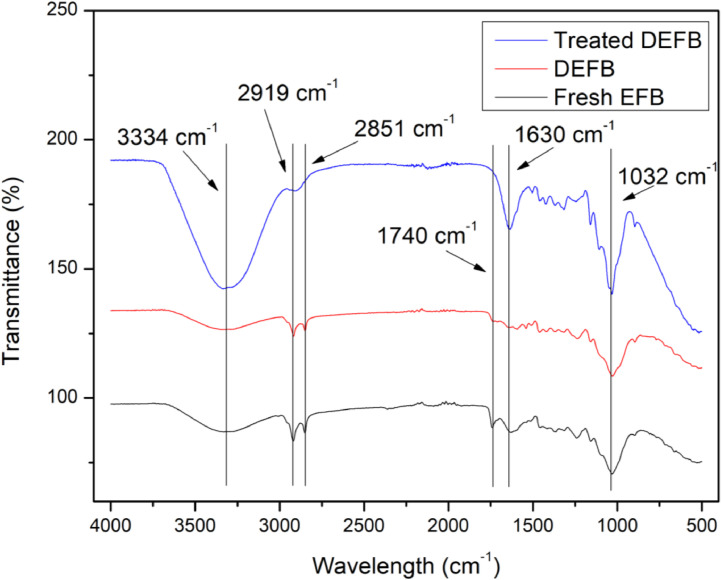
FTIR Spectrum of (a) treated DEFB after alkaline ethylene glycol pretreatment, (b) untreated DEFB and (c) untreated fresh EFB from 500 cm^−1^ to 4000 cm^−1^.

**Fig. 2:**
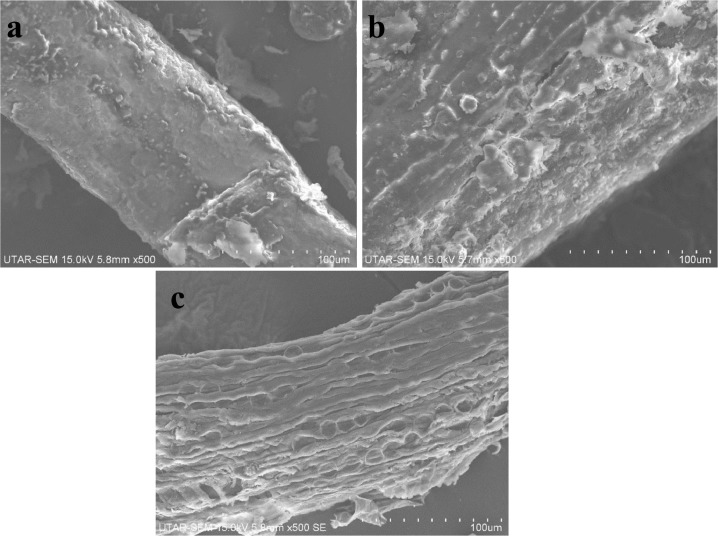
Surface morphology of (a) fresh EFB, (b) untreated DEFB, (c) treated DEFB after ethylene glycol pretreatment and (d) treated DEFB after ethylene glycol pretreatment with NaOH under 500  ×  magnification.

Fresh empty fruit bunch (EFB) is a lignocellulosic biomass waste which is produced during palm oil extraction process. Fresh EFB is highly susceptible to microbial attack when it is exposed to the open environment. The growth of the microorganisms will degrade its physical and chemical properties and affect its reutilisation. However, the degraded empty fruit bunch (DEFB) remains to be a potential source for biofuels and biochemical productions through alkaline ethylene glycol pretreatment and two-staged acid hydrolysis. In this study, different instrument characterisations were used to provide the physical and chemical information for the DEFB samples. Fig. 1 shows the FTIR spectrum of fresh EFB, DEFB and treated DEFB after the alkaline ethylene glycol pretreatment. Table 1 illustrates the XRD intensity of the similar samples after the alkaline ethylene glycol pretreatment. Fig. 2 shows their surface morphology while Table 2 lists the elemental compositions of different EFB samples. The validation of the analysis methods that were used to determine the product compositions of acid hydrolysate is also provided in Sections 2.3–2.5. Raw data of this article is available in the supplementary files.

**Table 1:** XRD peak intensities at 18.5^∘^ (amorphous region) and at 22.5^∘^ (crystalline region) of cellulose.

EFB samples	Intensity (I.U.)	
	18.5^∘^	22.5^∘^
Fresh EFB	116	369
Untreated DEFB	126	340
Alkaline ethylene glycol treated DEFB	154	418

**Table 2:** Elemental composition of fresh EFB, untreated DEFB and treated DEFB after alkaline ethylene glycol pretreatment.

Elemental composition (At%)	Fresh EFB	DEFB	Treated DEFB
Carbon	64.1	63.5	55.3
Oxygen	34.6	35.3	43.1
Sodium	0.1	0.1	1.0
Magnesium	0.2	0.3	0.2
Silicon	0.7	0.6	0.2
Phosphorous	0.1	0.1	0.1
Sulfur	0.1	0.1	0.1
Ferum	0.1	0.1	0.1

## 2 Experimental design, materials, and methods

### 2.1 Preparation of DEFB and alkaline organosolv pretreatment of DEFB

DEFB was collected from a plantation estate which is located at Segamat, Malaysia. Prior to utilisation, DEFB was separated into smaller pieces and dried at 100 °C overnight. The dried DEFB was subsequently grinded into powder form and sieved using the size of 850μm [2]. Predetermined amount of powdered DEFB was treated with 50 v/v% ethylene glycol in the presence of 3 v/v% NaOH at 80 °C for 45 min under 10 w/v% mass loading. Treated DEFB was washed thoroughly with distilled water for several times and dried in oven at 100 °C for overnight. Fresh EFB, untreated powdered DEFB and treated DEFB were subjected to characterisation analysis including FTIR, XRD, SEM and EDX as shown in Fig. 1, Table 1, Fig. 2 and Table 2, respectively. The FTIR spectrums of different EFB samples were obtained with Nicholet IS10 in the range of 500–4000 cm^−1^. XRD patterns were determined by using Shimadzu XRD-6000 from 5^∘^ to 60^∘^ at the speed of 2^∘^/min. The surface morphology with 500  ×  magnification was obtained with Hitachi SEM while elemental composition of sample was determined using Aimtek EDX.

### 2.2 Component analysis for two-staged acid hydrolysis

Treated DEFB was subjected to two-staged acid hydrolysis and the complete methodology can be obtained from our previous work [1].

### 2.3 DNS method for reducing sugars

**Fig. 3:**
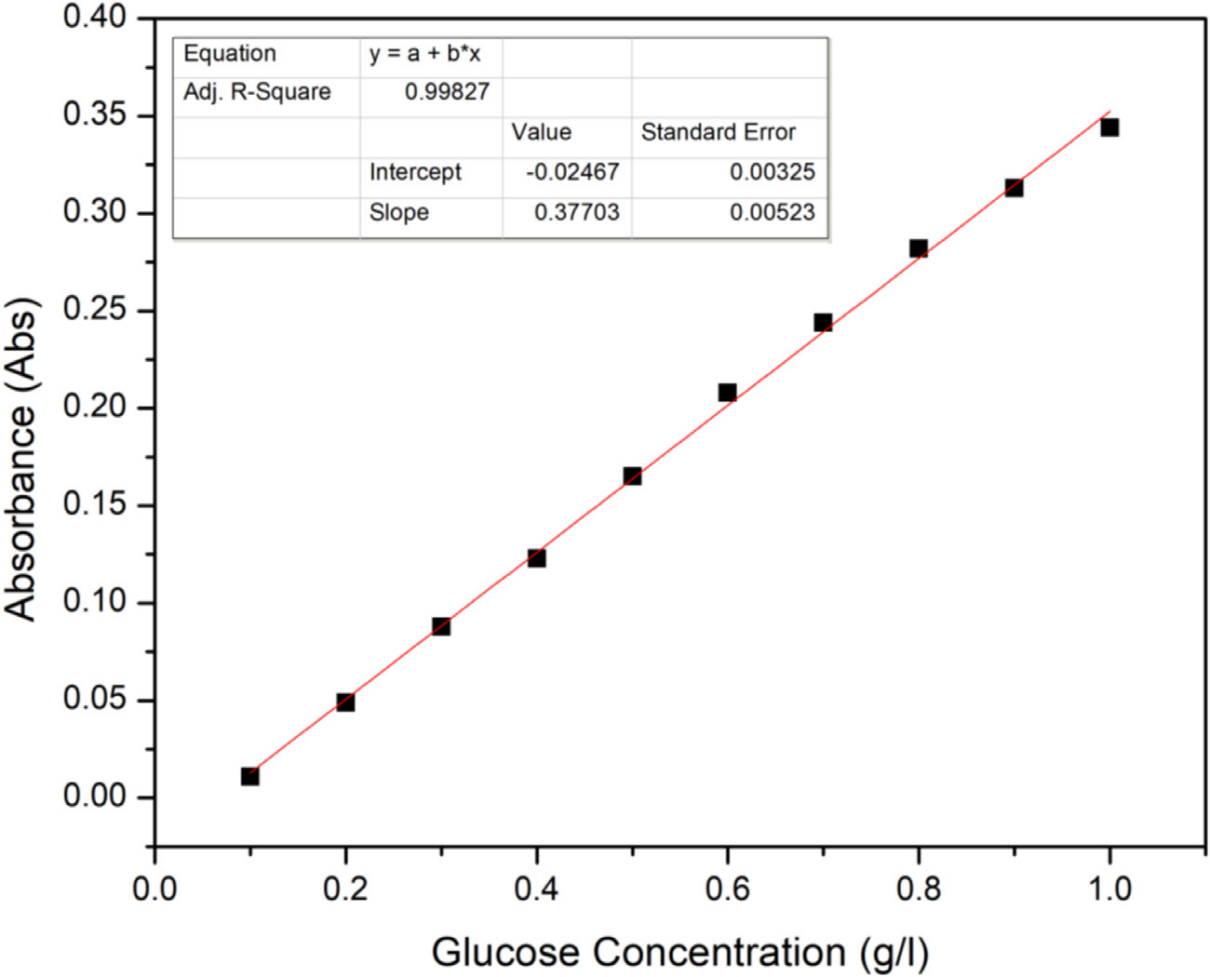
Calibration curve of DNS method by using glucose as standard at 540 nm detection.

DNS reagent was used to quantify the reducing sugars in acid hydrolysate [3]. 1 ml of the neutralised sample was added with 2 ml of 3,5-dinitrosalicylic acid (DNS) reagent before immersed in a water bath at 100 °C for 5 min. Afterwards, the solution was added with 7 ml of distilled water and allowed to cool down to room temperature. The solution was detected at 540 nm with UV–vis spectrophotometry (PG Instruments T-60) and glucose was used as the standard solution. The calibration curve of glucose and coefficient of determination (*R*^2^) are shown in Fig. 3.

### 2.4 Folin-Ciocaltues reagent method for phenolic contents

**Fig. 4:**
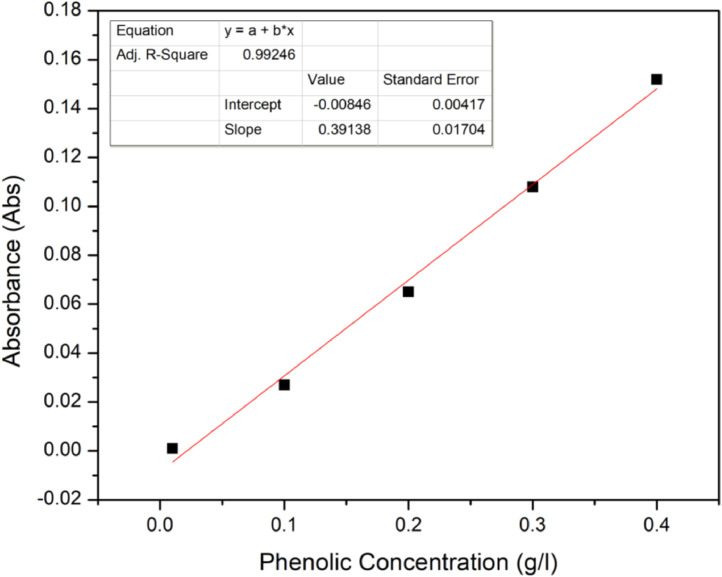
Calibration curve of phenolic compounds by using gallic acid as standard at 750 nm detection.

Folin-Ciocalteus reagent was used to determine the phenolic concentration in acid hydrolysate [4]. A total volume of 9 ml solution which included 8.4 ml distilled water, 0.5 ml Folin-Ciocalteus Reagent and 0.1 ml hydrolysate was prepared. The solution was allowed to react in the dark at room temperature for 1 h. The final solution was then subjected to UV–vis spectrophotometry (PG Instruments T-60) at 750 nm. The standard was prepared by using gallic acid and the calibration curve which recorded a *R*^2^ of 0.9924 is shown in Fig. 4.

### 2.5 HPLC method for furfural and HMF

**Fig. 5:**
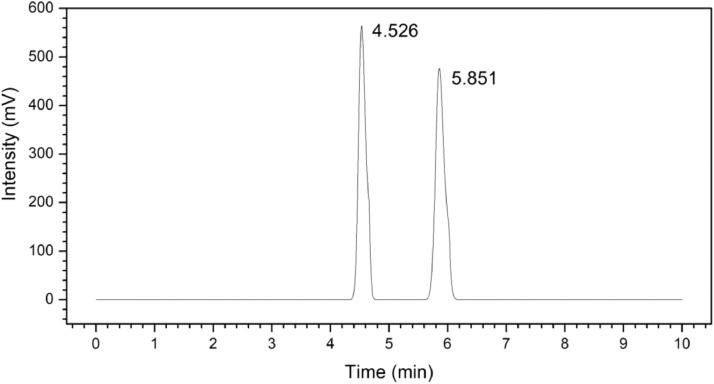
HPLC Chromatogram of furans (a) HMF (4.526 min) and (b) furfural (5.851 min) with Hypersil Gold C_18_ with flowrate 1.0 ml/min.

The quantifications of furfural and HMF were determined by using a reversed phase HPLC (Shimadzu LC-20AD) which was equipped with a quaternary pump unit, a column oven, auto-sampler and an UV–vis detector. Hypersil Gold C_18_ column (150 mm  ×  4.6 mm, 5 μm) was used to separate the furfural and HMF which were detected at 280 nm. The mobile phase was composed of 11 v/v% acetonitrile, 88 v/v% deionised water and 1 v/v% acetic acid [5]. The mobile phase was filtered with the membrane filtration for three times prior to its usage. The analysis was conducted at column temperature of 40 °C with fixed flowrate at 1.0 ml/min. The injection volume of sample was 10 μl. The HPLC chromatogram of HMF and furfural is shown in Fig. 5 and Table 3 shows the intraday and interday parameters of HMF and furfural.

**Table 3:** Intraday and Interday precision, retention time and limit of detection for HMF and furfural.

Components	Furfural	HMF
Retention time (min)	5.850	4.526
Intraday precision (%)		
Retention time	0.020	0.025
Peak area	0.586	0.285
Interday precision (%)		
Retention time	0.085	0.088
Peak area	1.043	0.312
Coefficient of determination (*R*^2^)	0.9999	0.9999

## Appendix Supplementary materials

Image, application 1

Image, application 2
