# Supplementary material for: Dataset of alkaline ethylene glycol pretreatment and two-staged acid hydrolysis using oil palm empty fruit bunch
Source: Data Brief. 2020 Mar 17;30:105431. doi: 10.1016/j.dib.2020.105431 (PMC7118301; doi:10.1016/j.dib.2020.105431)
Supplement: Supplementary file 1 [file mmc1.zip › Supplementary files/Table 3. HPLC/Raw Data for HPLC Detection of HMF and Furfural.docx]

**Raw Data for HPLC Detection of HMF and Furfural**

**Table S1 Interday and intraday rentention time of HMF**

| Retention Time (min) | Intraday | Interday |
| --- | --- | --- |
| R1 | 4.525 | 4.522 |
| R2 | 4.527 | 4.523 |
| R3 | 4.527 | 4.521 |

**Table S2 Interday and intraday rentention time of furfural**

| Retention Time (min) | Intraday | Interday |
| --- | --- | --- |
| R1 | 5.849 | 5.845 |
| R2 | 5.851 | 5.846 |
| R3 | 5.851 | 5.844 |

**Table S3 Interday and intraday rentention area under curve of HMF**

| Area under Curve | Intraday | Interday |
| --- | --- | --- |
| R1 | 7915044 | 7839490 |
| R2 | 7900840 | 7890123 |
| R3 | 7945001 | 7957045 |

**Table S4 Interday and intraday rentention area under curve of furfural**

| Area under Curve | Intraday | Interday |
| --- | --- | --- |
| R1 | 8085834 | 8174901 |
| R2 | 8051245 | 8106621 |
| R3 | 7992531 | 8099857 |

**Table S5 Calibration curve raw data of HMF and Furfural**

| Concentration (g/L) | HMF | Furfural |
| --- | --- | --- |
| 0.005 | 401400 | 415396 |
| 0.01 | 789450 | 815248 |
| 0.05 | 3984621 | 4118171 |
| 0.1 | 7915044 | 8174901 |
| 0.5 | 37966794 | 40514237 |
