# Supplementary figures and images for: Dataset of alkaline ethylene glycol pretreatment and two-staged acid hydrolysis using oil palm empty fruit bunch
Source: Data Brief. 2020 Mar 17;30:105431. doi: 10.1016/j.dib.2020.105431 (PMC7118301; doi:10.1016/j.dib.2020.105431)

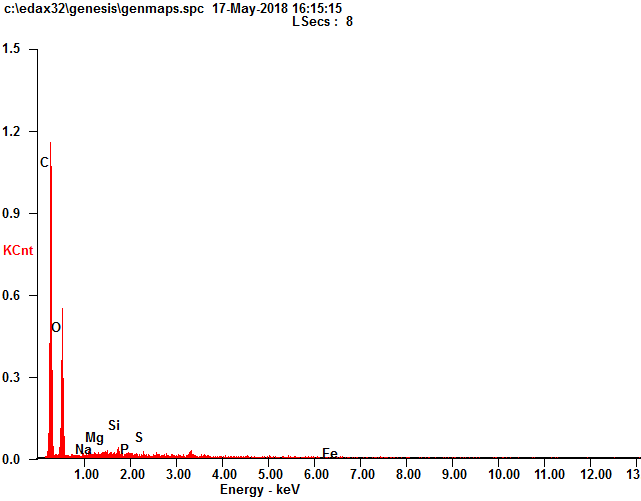


| ***Element*** | ***Wt%*** | ***At%*** |
| --- | --- | --- |
| ***CK*** | 56.87 | 64.00 |
| ***OK*** | 42.03 | 35.51 |
| ***NaK*** | 00.18 | 00.11 |
| ***MgK*** | 00.22 | 00.12 |
| ***SiK*** | 00.23 | 00.11 |
| ***PK*** | 00.00 | 00.00 |
| ***SK*** | 00.16 | 00.07 |
| ***FeK*** | 00.30 | 00.07 |
| ***Matrix*** | Correction | ZAF |


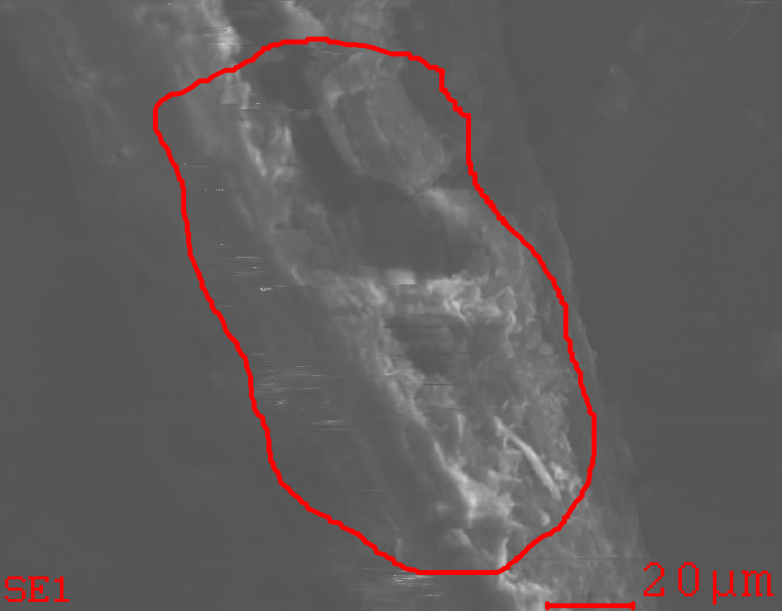

Supplement: Supplementary file 1 [file mmc1.zip › Supplementary files/Table 2. EDX/Fresh EFB (1).docx]

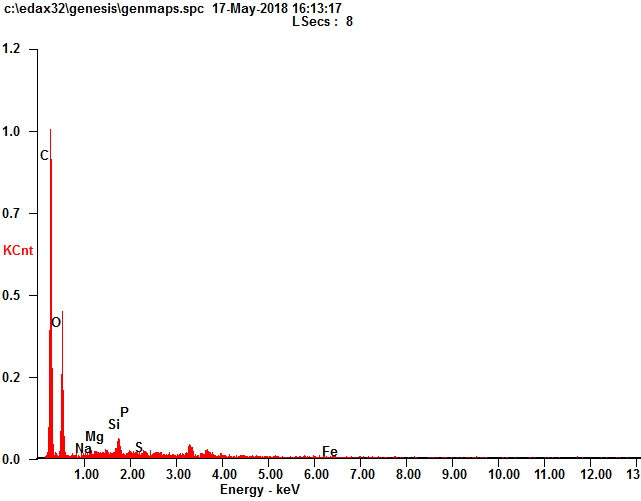


| ***Element*** | ***Wt%*** | ***At%*** |
| --- | --- | --- |
| ***CK*** | 59.44 | 66.95 |
| ***OK*** | 37.37 | 31.60 |
| ***NaK*** | 00.30 | 00.18 |
| ***MgK*** | 00.35 | 00.20 |
| ***SiK*** | 01.35 | 00.65 |
| ***PK*** | 00.24 | 00.11 |
| ***SK*** | 00.49 | 00.21 |
| ***FeK*** | 00.45 | 00.11 |
| ***Matrix*** | Correction | ZAF |


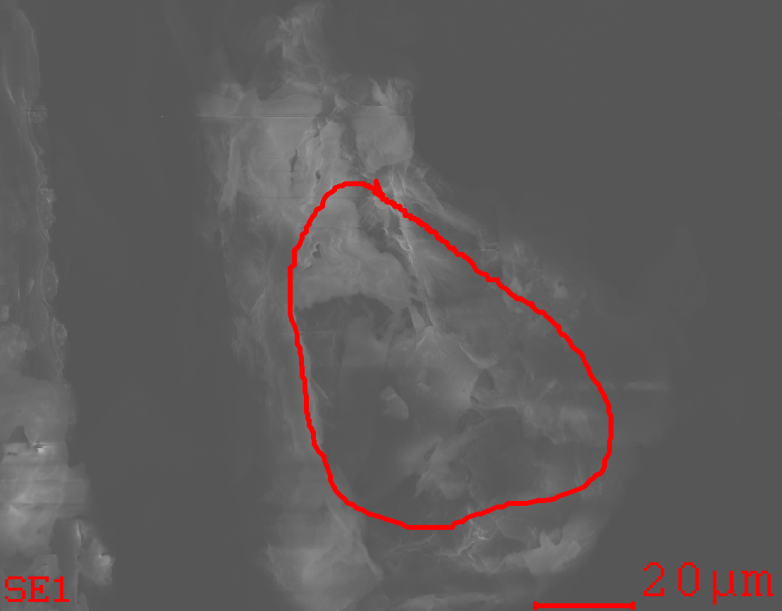

Supplement: Supplementary file 1 [file mmc1.zip › Supplementary files/Table 2. EDX/Fresh EFB (2).docx]

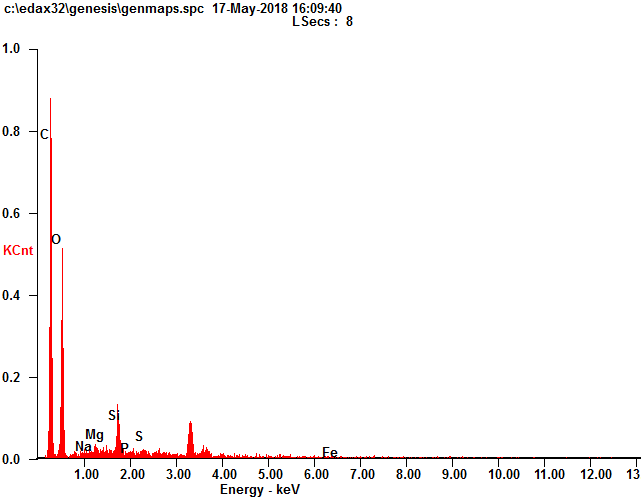


| ***Element*** | ***Wt%*** | ***At%*** |
| --- | --- | --- |
| ***CK*** | 53.44 | 61.41 |
| ***OK*** | 42.37 | 36.56 |
| ***NaK*** | 00.12 | 00.07 |
| ***MgK*** | 00.63 | 00.36 |
| ***SiK*** | 02.72 | 01.34 |
| ***PK*** | 00.20 | 00.09 |
| ***SK*** | 00.26 | 00.11 |
| ***FeK*** | 00.27 | 00.07 |
| ***Matrix*** | Correction | ZAF |


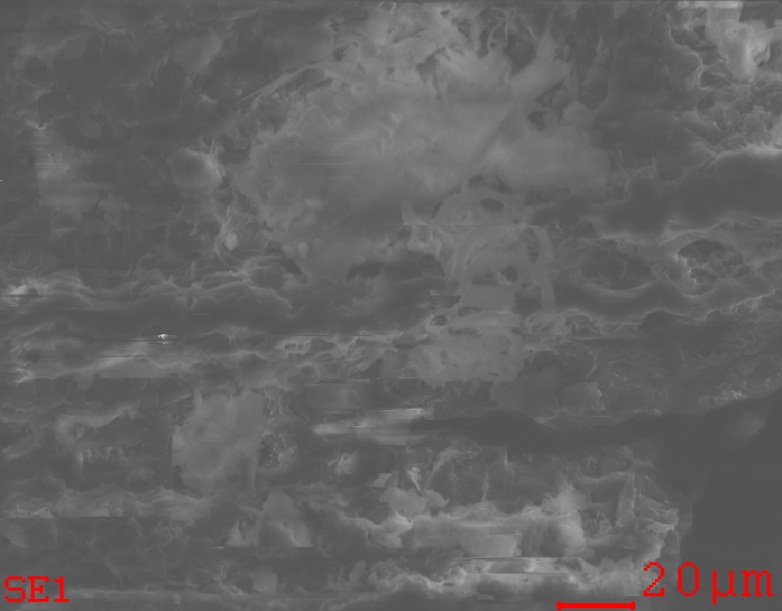

Supplement: Supplementary file 1 [file mmc1.zip › Supplementary files/Table 2. EDX/Fresh EFB (3).docx]

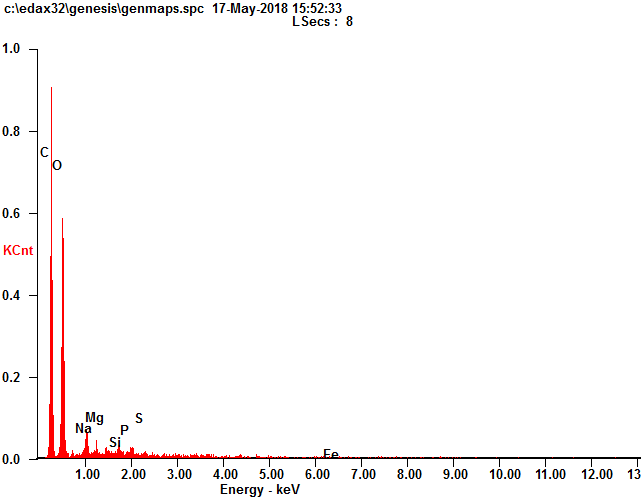


| ***Element*** | ***Wt%*** | ***At%*** |
| --- | --- | --- |
| ***CK*** | 47.11 | 55.04 |
| ***OK*** | 48.39 | 42.44 |
| ***NaK*** | 02.58 | 01.58 |
| ***MgK*** | 00.63 | 00.37 |
| ***SiK*** | 00.49 | 00.25 |
| ***PK*** | 00.57 | 00.26 |
| ***SK*** | 00.12 | 00.05 |
| ***FeK*** | 00.10 | 00.02 |
| ***Matrix*** | Correction | ZAF |


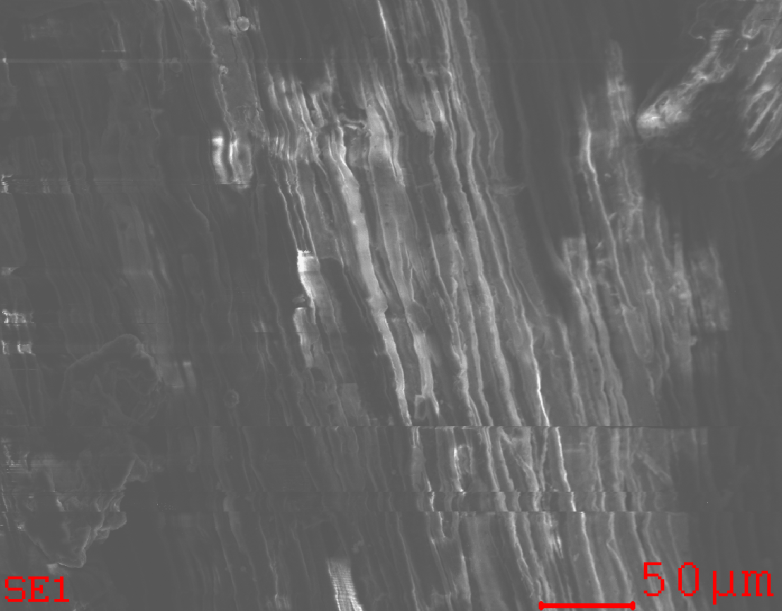

Supplement: Supplementary file 1 [file mmc1.zip › Supplementary files/Table 2. EDX/Treated DEFB (1).docx]

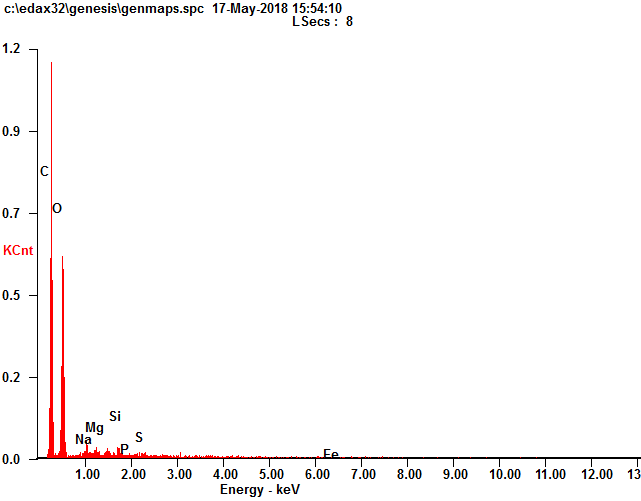


| ***Element*** | ***Wt%*** | ***At%*** |
| --- | --- | --- |
| ***CK*** | 49.62 | 57.23 |
| ***OK*** | 47.64 | 41.25 |
| ***NaK*** | 01.35 | 00.82 |
| ***MgK*** | 00.46 | 00.26 |
| ***SiK*** | 00.61 | 00.30 |
| ***PK*** | 00.11 | 00.05 |
| ***SK*** | 00.20 | 00.09 |
| ***FeK*** | 00.00 | 00.00 |
| ***Matrix*** | Correction | ZAF |


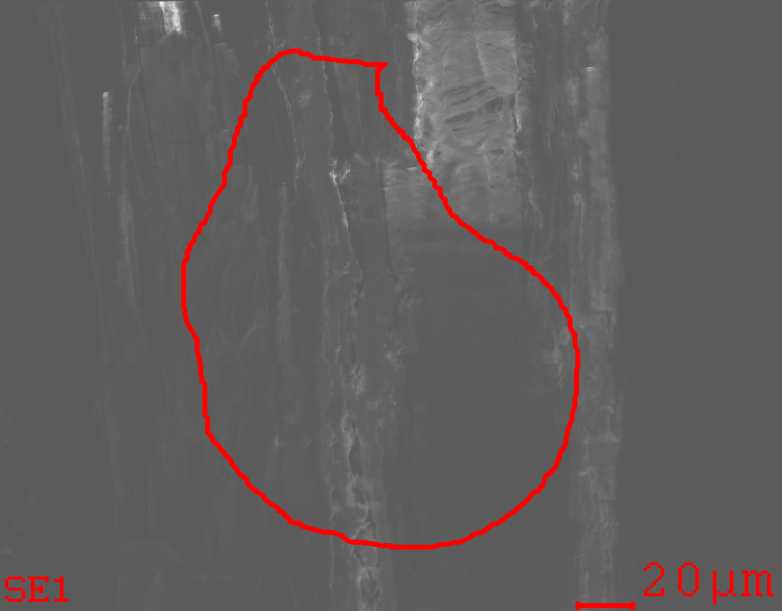

Supplement: Supplementary file 1 [file mmc1.zip › Supplementary files/Table 2. EDX/Treated DEFB (2).docx]

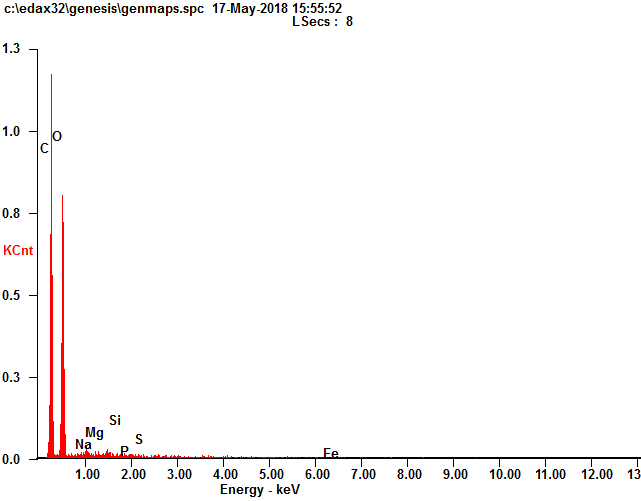


| ***Element*** | ***Wt%*** | ***At%*** |
| --- | --- | --- |
| ***CK*** | 46.28 | 53.67 |
| ***OK*** | 52.41 | 45.62 |
| ***NaK*** | 00.75 | 00.46 |
| ***MgK*** | 00.20 | 00.11 |
| ***SiK*** | 00.06 | 00.03 |
| ***PK*** | 00.13 | 00.06 |
| ***SK*** | 00.10 | 00.04 |
| ***FeK*** | 00.07 | 00.02 |
| ***Matrix*** | Correction | ZAF |


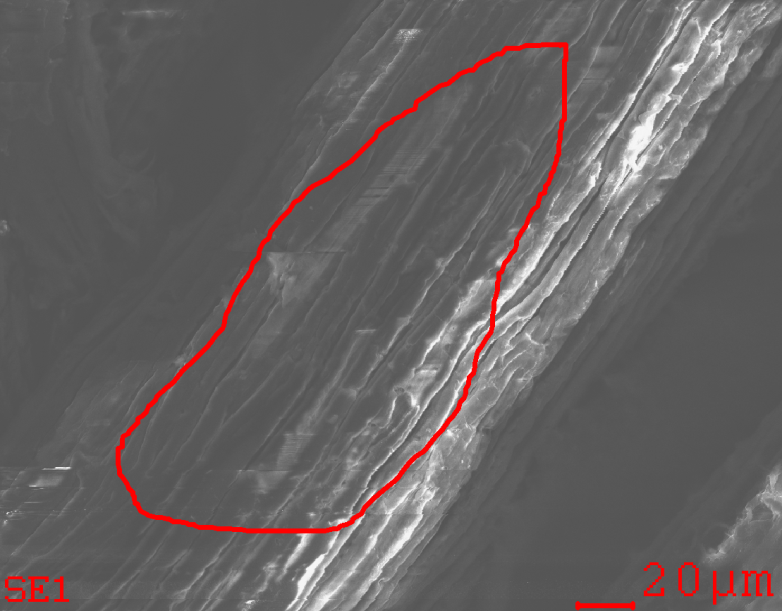

Supplement: Supplementary file 1 [file mmc1.zip › Supplementary files/Table 2. EDX/Treated DEFB (3).docx]

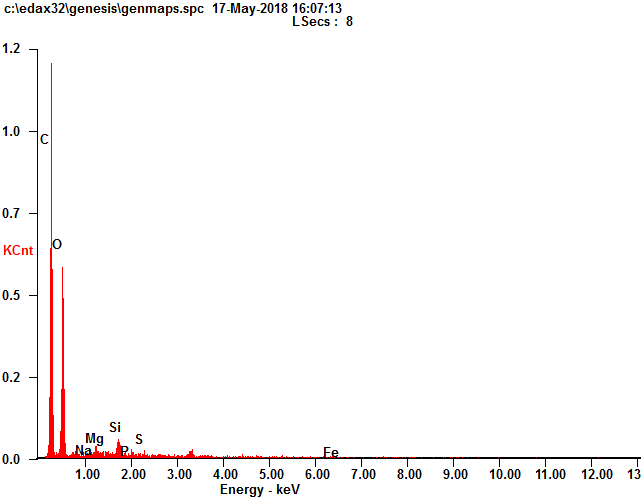


| ***Element*** | ***Wt%*** | ***At%*** |
| --- | --- | --- |
| ***CK*** | 52.92 | 60.49 |
| ***OK*** | 44.73 | 38.39 |
| ***NaK*** | 00.09 | 00.05 |
| ***MgK*** | 00.50 | 00.28 |
| ***SiK*** | 01.09 | 00.53 |
| ***PK*** | 00.26 | 00.11 |
| ***SK*** | 00.19 | 00.08 |
| ***FeK*** | 00.23 | 00.06 |
| ***Matrix*** | Correction | ZAF |


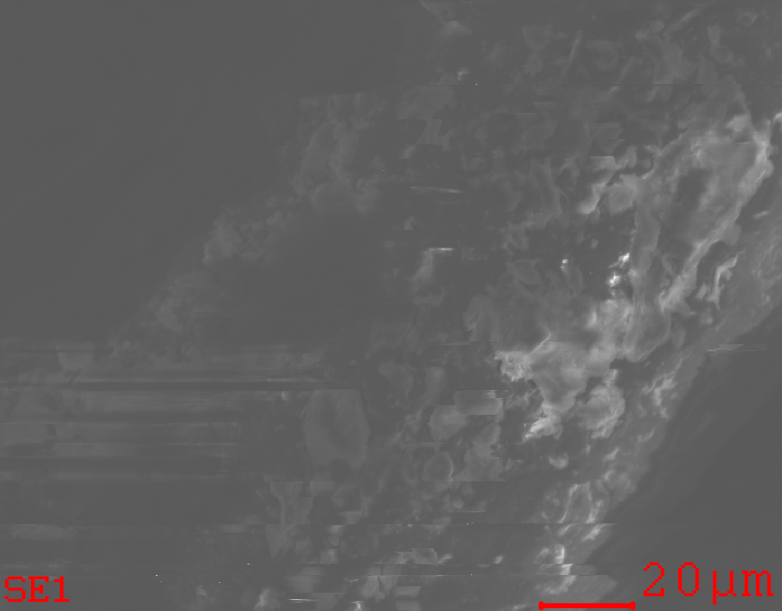

Supplement: Supplementary file 1 [file mmc1.zip › Supplementary files/Table 2. EDX/Untreated DEFB (1).docx]

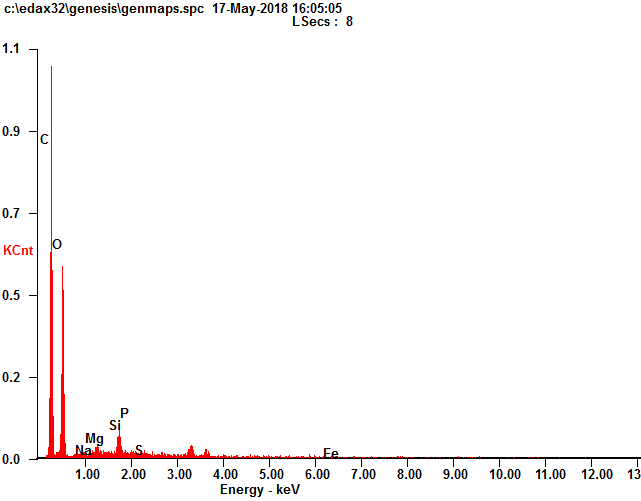


| ***Element*** | ***Wt%*** | ***At%*** |
| --- | --- | --- |
| ***CK*** | 53.42 | 61.24 |
| ***OK*** | 43.28 | 37.25 |
| ***NaK*** | 00.00 | 00.00 |
| ***MgK*** | 00.60 | 00.34 |
| ***SiK*** | 01.75 | 00.86 |
| ***PK*** | 00.19 | 00.08 |
| ***SK*** | 00.25 | 00.11 |
| ***FeK*** | 00.52 | 00.13 |
| ***Matrix*** | Correction | ZAF |


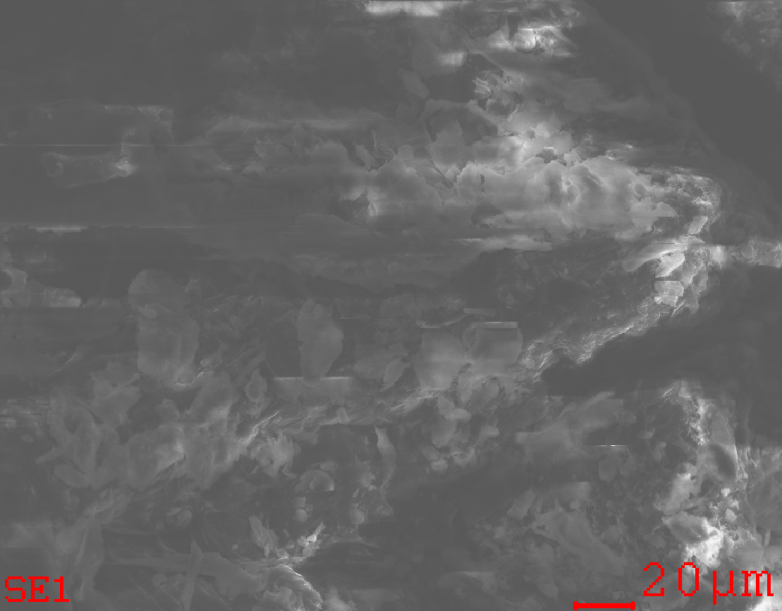

Supplement: Supplementary file 1 [file mmc1.zip › Supplementary files/Table 2. EDX/Untreated DEFB (2).docx]

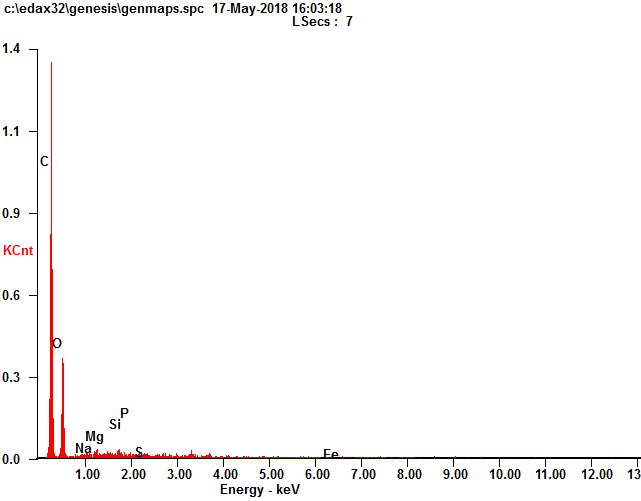


| ***Element*** | ***Wt%*** | ***At%*** |
| --- | --- | --- |
| ***CK*** | 61.60 | 68.66 |
| ***OK*** | 36.16 | 30.26 |
| ***NaK*** | 00.40 | 00.23 |
| ***MgK*** | 00.54 | 00.30 |
| ***SiK*** | 00.67 | 00.32 |
| ***PK*** | 00.19 | 00.08 |
| ***SK*** | 00.20 | 00.08 |
| ***FeK*** | 00.24 | 00.06 |
| ***Matrix*** | Correction | ZAF |


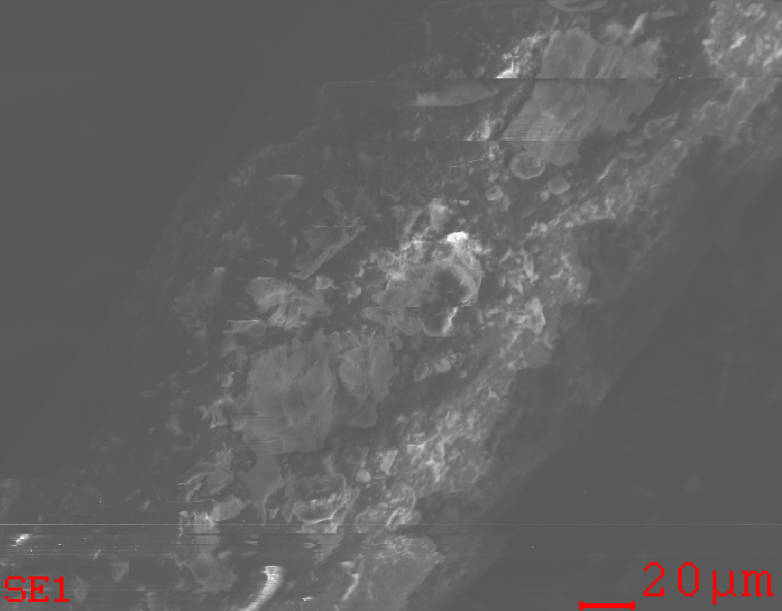

Supplement: Supplementary file 1 [file mmc1.zip › Supplementary files/Table 2. EDX/Untreated DEFB (3).docx]
